# Pulmonary Rehabilitation in SARS-CoV-2: A Systematic Review and Meta-Analysis of Post-Acute Patients

**DOI:** 10.3390/diagnostics12123032

**Published:** 2022-12-02

**Authors:** Glenn Reinert, Daniel Müller, Pit Wagner, Oliver Martínez-Pozas, Juan Nicolás Cuenca-Záldivar, Josué Fernández-Carnero, Eleuterio A. Sánchez Romero, Camilo Corbellini

**Affiliations:** 1 Hopitaux Robert Schuman-Centre Médical Clinique Sainte Marie, Rue Wurth-Paquet 7, 4350 Esch-sur-Alzette, Luxembourg; glenn.reinert1990@gmail.com; 2 Physiotherapy Masters of Science Programme, LUNEX International University of Health, Exercise and Sports, 50, Avenue du Parc des Sports, 4671 Differdange, Luxembourg; mueller.daniel@chl.lu (D.M.); pit_wagner@hotmail.com (P.W.); 3 Physiotherapy Unit, Centre Hospitalier de Luxembourg, Rue Ernest Barble 4, 1210 Luxembourg, Luxembourg; 4 Escuela Internacional de Doctorado, Department of Physical Therapy, Occupational Therapy, Rehabilitation and Physical Medicine, Universidad Rey Juan Carlos, 28933 Alcorcón, Spain; 5 Research Group in Nursing and Health Care, Puerta de Hierro Health Research Institute—Segovia de Arana (IDIPHISA), 28222 Majadahonda, Spain; 6 Physical Therapy Unit, Primary Health Care Center “El Abajón”, 28231 Las Rozas de Madrid, Spain; 7 Department of Physical Therapy, Occupational Therapy, Rehabilitation and Physical Medicine, Universidad Rey Juan Carlos, 28922 Alcorcón, Spain; josue.fernandez@urjc.es; 8 Musculoskeletal Pain and Motor Control Research Group, Faculty of Sport Sciences, Universidad Europea de Madrid, 28670 Madrid, Spain; 9 Musculoskeletal Pain and Motor Control Research Group, Faculty of Health Sciences, Universidad Europea de Canarias, 38300 Santa Cruz de Tenerife, Spain; 10 Department of Physiotherapy, Faculty of Sport Sciences, Universidad Europea de Madrid, 28670 Villaviciosa de Odón, Spain; 11 Department of Physiotherapy, Faculty of Health Sciences, Universidad Europea de Canarias, 38300 Santa Cruz de Tenerife, Spain; 12 Department of Physiotherapy, LUNEX International University of Health, Exercise and Sports, 50, Avenue du Parc des Sports, 4671 Differdange, Luxembourg; camilo.corbellini@lunex-university.net

**Keywords:** COVID-19, pulmonary rehabilitation, dyspnea, physical function, quality of life

## Abstract

Background: Pulmonary Rehabilitation (PR) was initially developed for the management of Chronic Obstructive Pulmonary Disease (COPD) and is now recognized as a core management of COVID-19 patients. This systematic review and meta-analysis examined the efficacy of PR in patients with post-acute COVID-19 infection. Methods: A literature search was conducted in PubMed, the Web of Science (WoS), and the Cochrane Library from their inceptions until October 2022, and randomized controlled trials and observational studies were considered. The outcomes measured included dyspnea, physical function, and quality of life. Results: Eleven studies including 677 participants with post-acute COVID-19 were included in this analysis. From a qualitative point of view and analyzing the studies separately, PR improves dyspnea, physical function, and quality of life in patients with post-acute COVID-19. However, in pooling the data of all the studies, no significant changes pre-postintervention, compared to the control, were found among the experimental studies included in the analysis in any outcome measures, due to the high heterogeneity between the studies, as well as no significant improvements being found in the observational studies. A subgroup analysis revealed significant differences in all the included outcomes. Future studies should include the same scale to assess the actual efficacy of PR. Conclusion: From a qualitative analysis point of view, PR is effective in improving physical function, reducing dyspnea, and improving quality of life in patients with post-acute COVID-19. However, an exploratory meta-analysis was performed to evaluate, by subgroups, the efficacy of PR, and positive results were found in favor of PR.

## 1. Introduction

In late 2019, a novel, highly infectious virus emerged, causing a global pandemic [1]. The data sources from the World Health Organization report more than 400 million infections and more than 5.7 million deaths [2]. Although SARS-CoV-2 (COVID-19) predominantly affects the respiratory system, it also causes chronic pain, neurological disorders, musculoskeletal disorders, depression, anxiety, impaired physical function, and impaired quality of life (QoL), indicating evidence of a multisystem disease [3,4]. Those clinical and functional manifestations were observed to different degrees, starting from asymptomatic patients, followed by patients in isolation, then patients with a hospital stay, and, finally, patients at a critical life-threatening stage in the intensive care unit (ICU) [5,6,7].

Hypertension, coronary artery disease, chronic obstructive pulmonary disease (COPD), stroke, and diabetes have been detected as leading comorbidities [7,8]. Hence, pre-existing cardiovascular and cerebrovascular diseases may influence the rehabilitation outcome and duration [9]. A high prevalence of respiratory function impairment is strongly linked to pathophysiological events, such as diffuse alveolar epithelium destruction, hyaline membrane formation, alveolar septal fibrous proliferation, capillary damage, and bleeding, as well as pulmonary consolidation, and reveals that an impairment of diffusion capacity, followed by restrictive ventilatory defects, are the most common abnormalities of lung function [10,11,12]. Pulmonary capillary destruction and pulmonary vasoconstriction result in pulmonary hypertension and cardiac dysfunction in some patients [13,14].

Pulmonary Rehabilitation (PR) was initially developed for the management of COPD and is now recognized as core management of various chronic cardiopulmonary conditions [5,6,8,15]. PR includes a patient assessment, regular participation in an individual exercise program, and patient educational and behavioral change [16,17]. A fundamental segment of PR is endurance training, consisting of walking, cycling, or a combination of both [18,19]. Another component of PR is resistance training to enhance muscle mass and strength, specifically for the peripheral muscles, and flexibility training to improve thoracic mobility and posture [19,20,21,22] PR is a safe treatment with no adverse effects, reducing dyspnea, increasing exercise tolerance and quality of life. PR has caused a significant reduction in the rate and duration of hospital admissions in patients with restrictive lung disease [23,24].

Detailed patient information on clinical outcomes to demonstrate the effectiveness of PR in patients with COVID-19 is lacking. Therefore, only a few studies are available to include in terms of the desired clinical outcomes [25]. Large sample size variations and the study design may be responsible for the high heterogeneity of the studies’ statistics. In addition, not all the studies compare the comorbidities of severe and non-severe patients. The different duration of the follow-up period may also influence the heterogeneity [26].

In 2020, a systematic review and meta-analysis were conducted to assess the efficacy of PR in COVID-19 patients and found that PR was effective and superior to no intervention in patients with COVID-19 [27]. However, from 2020 to today, new studies have been published, and there is a need to update the available scientific evidence. Additionally, another recent systematic review found that telerehabilitation may improve dyspnea and physical function in patients with post-acute COVID-19, but it only assessed telerehabilitation and excluded in-person treatments [28]. 

Therefore, the main aim of this systematic review with a meta-analysis is to assess the efficacy of PR (in-person or telerehabilitation) in patients with post-acute COVID-19. 

## 2. Materials and Methods

The methods for this systematic review followed the principles of the Preferred Reporting Items for Systematic Reviews and Meta-Analyses statement (PRISMA) [29]. Furthermore, the protocol was registered with the OSF (https://doi.org/10.17605/osf.io/g2vcp, accessed on 4 October 2022).

### 2.1. Eligibility Criteria

The eligible studies included randomized controlled trials (RCTs) and observational studies. The studies were selected if they met the criteria for assessing the existing evidence on PR in COVID-19 patients. The inclusion of telerehabilitation as part of PR was due to the progression of SARS-CoV-2 disease and the reconsideration of conventional means. The studies with a publication period of 2021–2022 were considered. Articles written in English, French, German, Spanish, Portuguese, or Italian, or translated into English, were considered.

### 2.2. Outcome Measures

The outcome measures considered to assess the effectiveness of PR in post-acute patients with COVID-19 were those related to physical function, dyspnea, and quality of life.

### 2.3. Data Sources and Search Strategy

The following databases were searched: PubMed, the Web of Science (WoS), and the Cochrane Library. The reference lists of the eligible articles identified during the search were manually searched. The PubMed search string was the following: ((COVID OR COVID-19 OR SARS-CoV-2 OR Coronavirus) AND (Pulmonary rehabilitation OR rehabilitation OR physical therapy OR exercise)) and were restricted to RCTs and observational studies. For the other databases, the string was modified if needed. No date restrictions were applied. 

The database search took place in November 2021 and was updated in October 2022 to fully review the current literature.

### 2.4. Data Screening and Extraction

Five independent researchers (GR, PW, DM, OMP, and EASR) reviewed the titles and abstracts for eligibility based on the criteria mentioned above. The five reviewers had independent access to the used platforms and discussed or tried to reach a consensus on the eligibility of the article in case of disagreement. This process was repeated when the full-text articles were reviewed. The five reviewers independently screened the full-text articles and decided which to include. After the inclusion of the studies, the reviewers extracted the appropriate data from the texts. The data on the study design, population and sample size, detailed intervention, detailed control, and reported findings were independently extracted by the reviewers.

### 2.5. Quality Assessment

The risk of bias in the RCTs was assessed using Cochrane’s Risk of Bias tool 2.0 (RoB 2.0) for randomized clinical trials [30]. This tool evaluates the randomization process, deviations from the intended interventions, missing outcome data, measurement of outcomes, and selection of the reported results, classifying studies into a low risk, some concerns, and a high risk of bias.

When the observational studies were evaluated, the Newcastle–Ottawa Scale (NOS) was used to assess the methodological quality [31]. The NOS assesses the quality of studies based on three domains: selection (4 items), comparability (1 item), and outcomes (3 items) [32]. The “selection” and “outcome” domains scored from 0 to 1, and the “comparability” domain scored from 0 to 2; the total score ranged from 0 to 9, with the higher scores indicating better quality. The studies were grouped into good quality (>7/9 points), fair quality (>5–7/9), and low quality (0–4/9), as were the previous studies [33,34].

Two independent researchers (EASR and OMP) assessed the methodological quality and risk of bias. In addition, we calculated the kappa coefficient (κ) and the percentage of agreement scores to assess the reliability prior to any consensus. The inter-rater reliability was estimated using κ > 0.7, indicating a high level of agreement between the reviewers, κ of 0.5–0.7, indicating a moderate level of agreement, and κ < 0.5, a low level of agreement [35,36].

### 2.6. Certainty of Evidence

The certainty of the evidence analysis was established by the different levels of evidence according to the Grading of Recommendations, Assessment, Development, and Evaluation (GRADE) framework, which is based on five domains: study design, imprecision, indirectness, inconsistency, and publication bias [37]. The evidence was classified into the following four levels: high quality (all domains satisfied), moderate (one domain not satisfied), low quality (two domains not satisfied), or very low quality (three or more domains not satisfied) [38].

For the risk of bias domain, the recommendations were downgraded one level if there was an unclear or high risk of bias and severe limitations on the estimation effect. For consistency, the recommendations were downgraded when the point estimates varied widely among the studies, the confidence intervals overlapped, or when the I^2^ test was substantial (>50%). For the indirectness domain, when significant differences in interventions, populations, or outcomes were found, the recommendations were downgraded by one level. If there were fewer than 300 participants for the key outcomes in the imprecision domain, they were downgraded by one level. Finally, if a strong influence of publication bias was detected, the recommendations were downgraded by one level [39].

### 2.7. Data Synthesis

For the statistical analysis, the R Ver. 4.1.3 program was used (R Foundation for Statistical Computing, Institute for Statistics and Mathematics, Welthandelslplatz 1, 1020, Vienna, Austria).

In the articles in which the results were shown using the median, and with the maximum and minimum, these were transformed into the mean and standard deviation using the appropriate formulae [40,41].

In the RCTs, a meta-analysis of the pre-postintervention changes was performed by analyzing the level of significance between the treatment and control groups using the standardized mean difference (SMD). In the studies in which the data were not reported, they were calculated with the pre-post intervention data and the standard deviation of the change was determined using the formula [42]:

$$SD_{change}=\sqrt{SD_{baseline}^{2}+SD_{final}^{2}-\left(2\cdot r\cdot SD_{baseline}\cdot SD_{final}\right)},$$

where (*SD*) is the standard deviation and *r* is the pre-postintervention correlation coefficient obtained according to the formula:

$$r=\frac{SD_{baseline}^{2}+SD_{final}^{2}-SD_{change}^{2}}{2\cdot SD_{baseline}\cdot SD_{final}}.$$

The average of *r* was imputed in the missing data. In cases where these data were not available, the corresponding authors of the studies were asked for pre-postintervention and/or change data. Finally, when none of the required data could be obtained but the pre-post intervention standard deviation was available, a value of 0.7 was assigned to *r* in order to obtain a conservative estimate [43], as has been done in other studies [44,45,46]. For the observational studies, a single-group meta-analysis was performed using the pre-post intervention change mean in each study.

In both cases, a random effects model was applied, given the heterogeneity between the studies. The heterogeneity was analyzed by estimating the between-study variance τ^2^ (calculated with the DerSimonian–Laird estimator with the Hartung–Knapp correction), Cochran’s Q test, and the I2 estimator with the heterogeneity defined as non-important (<30%), moderate (30–50%), large (50–75%), and important (>75%). The heterogeneity was assessed using a sensitivity analysis with the leave-one-out method. Subgroup meta-analyses were also performed to explore the heterogeneity detected, depending on the type of test used in each of the three outcomes variables. The effect size was calculated in RCTs with Hedge’s g defined as small (<0.2), moderate (0.2–0.8), and large (>0.8). Finally, the publication bias was analyzed using trim and fill funnel plots [47] and the Begg and Egger tests.

## 3. Results

**Figure 1:**
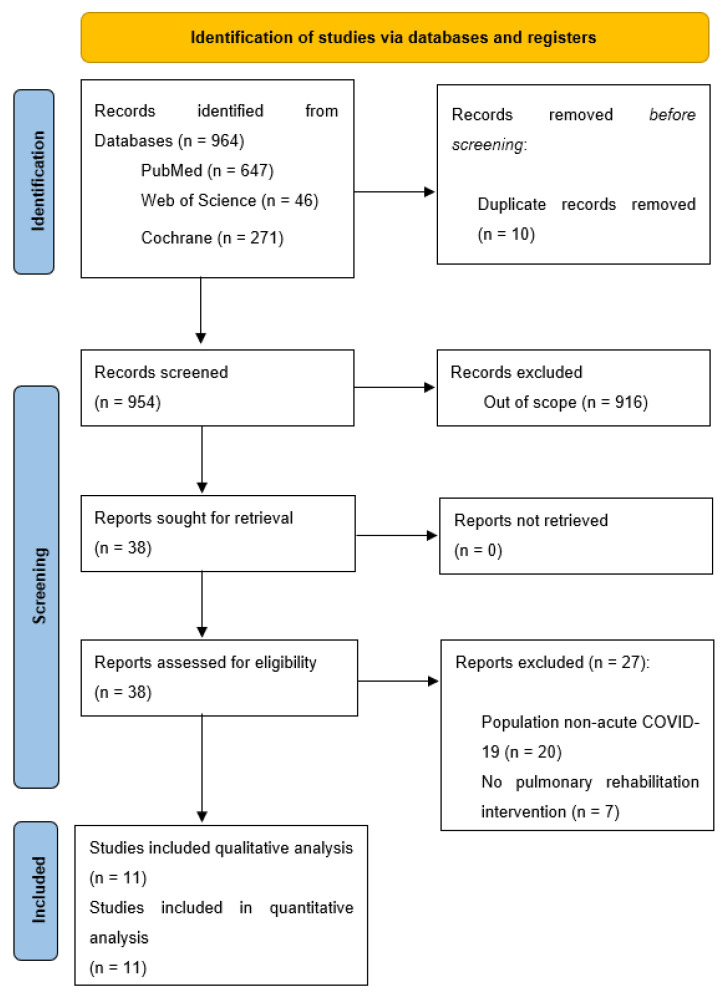
PRISMA flow diagram.

The search for publications resulted in 964 articles, retrieved from different search engines. After clearing the duplicates, 954 articles remained. A title screening identified 38 eligible articles. After the full-text review, only 11 articles were retained for this systematic review, following the eligibility criteria set for this paper. The results are included in Figure 1, according to the guidelines [48].

### 3.1. Characteristics of the Included Studies

Of the 11 included studies, a total of 677 patients were included within the studies. In most of the studies (7/11), the male ratio was higher than the female, and the population ranged from mild to severe COVID-19 symptoms, treated as out- and inpatients. The interventions included in-person or home-training breathing exercises, in addition to other exercises, such as aerobic and strengthening exercises, or other therapies such as physical therapy. The outcomes included physical performance (6MWD, SPPB, TUG, 30-CST, 30STS, 1min-STS), dyspnea (NRS/mMRC, DSI, MBS, MD12, BS), or quality of life variables (SF-12, SGRQ, EQ-5D). For a complete description of the included studies, see Table 1.

The range of article types included in this review is broad. Six randomized controlled trials [49,50,51,52,53,54], three cohort studies [55,56,57], one observational study [58], and one case-control trial [59]. The 11 included studies were conducted in Spain [49], China [50], Brazil [51], Iran [52,53], Turkey [54], France [55], Germany [56], Belgium [57], Switzerland [58], and Saudi Arabia [59].

### 3.2. Methodological Quality and Risk of Bias of the Included Studies

**Figure 2:**
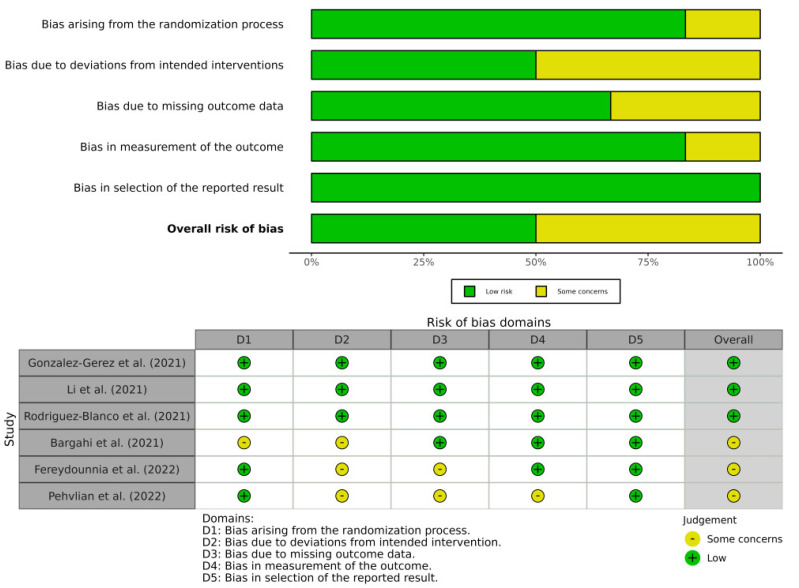
Risk of bias of RCTs assessed with RoB 2.0.

In total, six studies were evaluated. Only one of the included studies was evaluated as having a “low risk of bias” [49], three as “some concerns” [50,51,54], and two as a “high risk of bias” [52,53], suggesting that only 16% of the included randomized controlled trials have a low risk of bias. According to the domain analysis, the random sequencing and reporting of incomplete data had a low risk of bias in all the included studies, while the blinding participants and allocation concealment were the main risks of bias in the included studies. The inter-examiner (OMP and EASR) reliability had a high level of agreement (κ = 0.896). The risk of bias of the RCTs was assessed with the RoB 2.0 and the scores are shown in Figure 2 and Table 2.

The quality of the observational studies was evaluated with the Newcastle–Ottawa Scale and the scores are shown in Table 3.

In total, five studies were evaluated [55,56,57,58,59]. One study was evaluated as low quality [56], three as fair quality [55,57,58], and only one was evaluated as high quality [59]. The selection of controls (e.g., from the community), adjustment for confounding factors, and correct ascertaining of exposure were the main issues in the included studies. The inter-examiner (OMP and EASR) reliability had a high level of agreement (κ = 0.892).

### 3.3. Quality of Evidence

The quality of the evidence for pulmonary rehabilitation was assessed with the Grading of Recommendations, Assessment, Development, and Evaluation (GRADE) framework, and the results are shown in Table 4.

A very low quality of evidence supports the use of pulmonary rehabilitation to improve dyspnea, physical function, and quality of life in patients with post-acute COVID-19.

### 3.4. Data from Studies

The most relevant results obtained in the included studies are mentioned below.

#### 3.4.1. Effect of Pulmonary Rehabilitation on Dyspnea

Eight studies analyzed the effects of pulmonary rehabilitation on dyspnea [49,50,51,52,53,54,56,59]. To assess dyspnea, three studies used the modified Medical Research Council scale [50,54,56], four studies the Borg and Modified Borg Scale [49,51,52,53], one study the Dyspnea Severity Index [59], and the last one the Multidimensional Dyspnea 12 [49]. Six of the included studies were randomized controlled trials [49,50,51,52,53,54], and the risk of bias ranged from a low (16.6%), to unclear (49.8%) to a high risk of bias (33.6%). Two of included studies were observational [56,59], one of them of low quality [56], and the other one of high quality [59].

From a qualitative point of view, and analyzing each study separately, all the included studies reported improvements in dyspnea levels after pulmonary rehabilitation in patients with post-acute COVID-19 at the end of their treatments. When pulmonary rehabilitation was compared to the usual care, with no controls or educational instructions, statistically significant differences were found between the groups, favoring intervention in terms of dyspnea improvement [49,50,51,52,56]. However, when compared to general exercise, no significant differences were found [54]. Finally, the addition of myofascial release therapy to a pulmonary rehabilitation program, compared to pulmonary rehabilitation alone, resulted in statistical differences that favored the intervention group [53].

**Figure 3:**
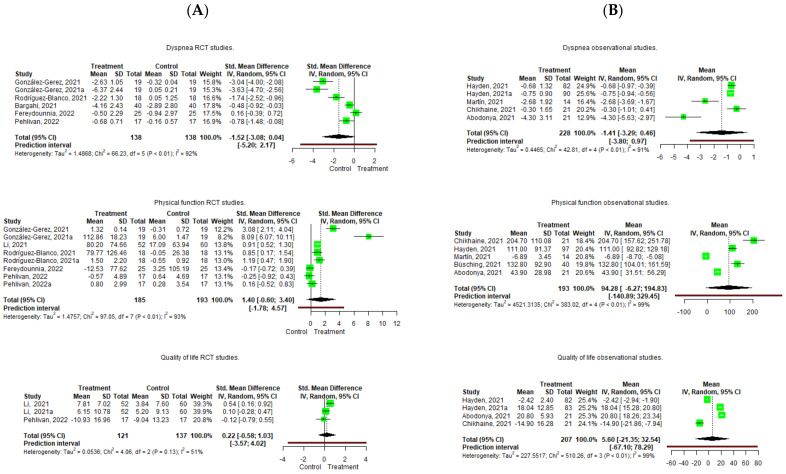
Forest plot of the effect of Pulmonary Rehabilitation in post-acute patients with COVID-19 related to physical function, dyspnea, and quality of life. (**A**) RCTs assessed; (**B**) Observational studies assessed.

Regarding the quantitative analysis, contrary results were found (Figure 3).

**Figure 4:**
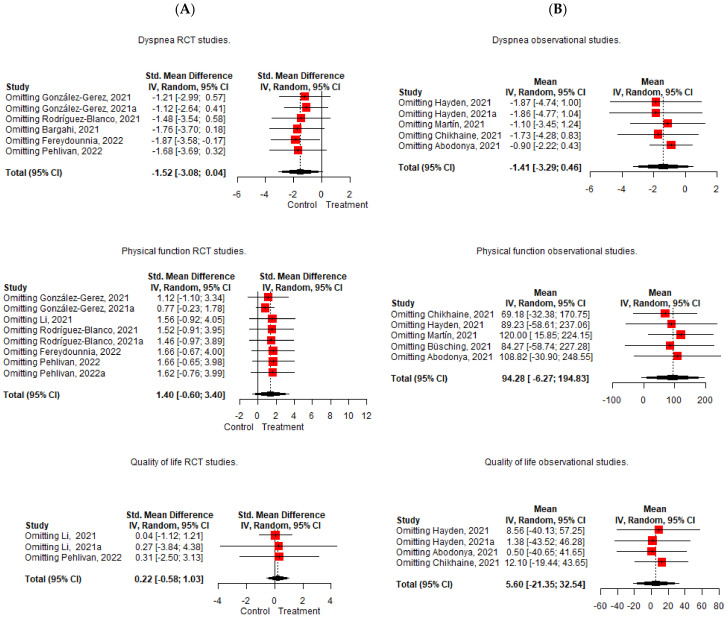
Leave-one-out analysis of the effect of Pulmonary Rehabilitation in post-acute patients with COVID-19 related to physical function, dyspnea, and quality of life. (**A**) RCTs assessed; (**B**) Observational studies assessed.

Pooling of the five included RCTs [49,51,52,53,54] did not result in significant effects on dyspnea improvement, with a lower pre-postintervention change in the treatment group (Hedge’s g = −1.517 95%, CI −3.076; 0.041, Z = −2.502, *p* = 0.054) and with an important heterogeneity (I^2^ = 92%). In case of the observational studies [55,56,57,59], the same scenario occurs, and no significant effects were observed in the pre-postintervention changes (mean = −1.414 95%, CI −3.292; 0.464) with an important degree of heterogeneity (I^2^ = 91%). The leave-one-out analysis shows that Fereydounnia et al. [53] and Abodonya et al. [59] were the most influential studies on the reduction of the effect on dyspnea [53,59] (Figure 4).

**Figure 5:**
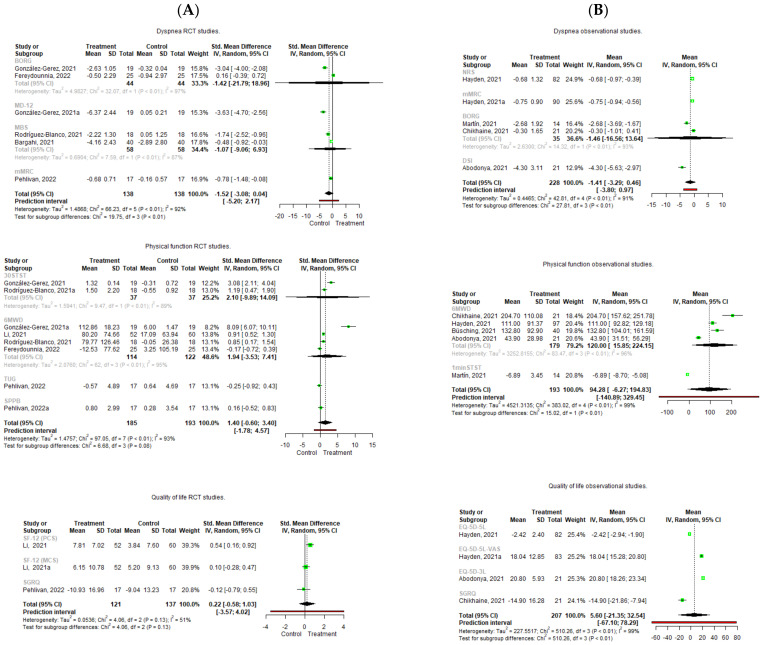
Subgroup meta-analysis of the effect of Pulmonary Rehabilitation in post-acute patients with COVID-19 related to physical function, dyspnea, and quality of life. (**A**) RCTs assessed; (**B**) Observational studies assessed.

When the meta-analysis of subgroups is carried out, the absence of significant effects is evident, except in some individual studies (Figure 5).

In the RCTs, the study by González-Gerez et al. [49] shows a significant and large effect with a higher increase in the MD-12 scale in the control group compared to the intervention group (Hedge´s g = −3.629 95%, CI 4.699; −2.56, Z = −6.654, *p* < 0.001). In the same way, the study of Pehlivan et al. [54] shows a significant and moderate effect with a higher increase in mMRC in the control group compared to the intervention group (Hedge´s g = −0.781 95%, CI −1.482; −0.081, Z = −2.185, *p* = 0.029). In the observational studies [55,56,57,59], the study of Hayden et al. [56] found statistically significant decreases in dyspnea levels after PR measured with the NRS (mean = −0.68 95%, CI −0.965; −0.395) and with a decrease in the mMRC score (mean = −0.75 95%, CI −0.935; −0.565). In the same way, Abodonya et al. [59] found statistically significant decreases in dyspnea measured with the DSI after PR (mean = −4.3 95%, CI −5.63; −2.97). However, heterogeneity remains at important values for both the RCTs [49,50,51,52,53,54] and observational studies [55,56,57,59] (only dyspnea measured with MBS I^2^ decreased from 92% to 87%).

#### 3.4.2. Effect of Pulmonary Rehabilitation on Physical Function

Ten studies analyzed the effects of pulmonary rehabilitation on physical function [49,50,51,53,54,55,56,57,58,59]. The 6 min walking test (6MWT) was the most common test to assess physical function and was used in five studies. Other studies used the 6 min walking distance test, Timed Up and Go Test, 1 min sit-to-stand test, 30 s sit-to-stand test (30-STS), or a short physical performance battery. Five of the included studies were randomized controlled trials and the risks of bias were low (20%), unclear (60%) and high (20%). Five observational studies were included and the methodological quality ranged from low (20%), to fair (40%), to high (40%).

Regarding the qualitative analysis, all the included studies reported improvements in physical performance after pulmonary rehabilitation at the end of the treatment in patients with post-acute COVID-19. When pulmonary rehabilitation is compared with the usual care or educational approaches, statistically significant differences favoring the intervention group in terms of physical function were found [49,50,51,57]. However, when compared to general exercise, there were no differences between the groups in terms of physical function improvements [54]. When manual therapy was added to a PR program, compared to PR alone, no significant differences were found between the groups [53].

Regarding the quantitative analysis, the pooling of five RCTs [49,50,51,53,54] found no significant effects on physical function improvement, with higher pre-postintervention changes in the treatment group compared to the control (Hedge´s g = 1.399 95%, CI −0.6; 3.397, Z = 1.655, *p* = 0.142), with an important heterogeneity (I^2^ = 93%). In the observational studies, five studies were pooled [55,56,57,58,59], and no significant effects were observed in the pre-postintervention change in physical performance (Mean = 94.282 95%, CI −6.26; 194.829), with important heterogeneity (I^2^ = 99%) (Figure 3). In the leave-one-out analysis, the study of Martin et al. [57] is the most influential study in the reduction of the effect (Figure 4).

When the subgroup analysis was performed separately (Figure 4), the 6MWD pooled data from the observational studies [55,56,58,59] showed significant increase scores after PR (mean = 0.537 95%, CI 0.159; 0.916), although important heterogeneity was found again (I^2^ = 96%). Additionally, Martin et al. [57] found a significant effect on physical performance with a reduction in the 1-min sit-to-stand test score (mean = −6.888 95%, CI −8.698; −5.078). The heterogeneity remains at important values and only decreases in the RCTs with the 30STST scale [49,51] (I^2^ of 93%, which goes down to 89%), and in the observational studies with the 6MWD scale [55,56,58] (I^2^ from 99% going down to 96%).

#### 3.4.3. Effect of Pulmonary Rehabilitation on Quality of Life

Five studies analyzed the effects of pulmonary rehabilitation on quality of life [50,54,55,56,59]. The EuroQol-5D was the most commonly used test, followed by the St George’s Respiratory Questionnaire and SF-12. Two of the included studies were RCTs [50,54] with an unclear risk of bias in both of them. The other three studies were observational, with the methodological quality classified as low [55], fair [56] and high [59] quality.

Regarding the qualitative analysis, all the included studies reported improvements in quality of life after pulmonary rehabilitation in patients with post-acute COVID-19. When pulmonary rehabilitation was compared to educational approaches, home exercises, or without (the control group), statistical differences favoring the intervention group were found [50,54,56]. One study found that adding inspiratory muscle training to a breathing exercise program resulted in improvements in quality of life compared to the breathing exercises alone [59].

Regarding the quantitative analysis, the pooling of two RCTs [50,54] showed a non-significant effect on quality of life, with a higher change in the treatment group (Hedge´s g = 0.224 95%, CI −0.582; 1.029, Z = −2.502, *p* = 0.354) and with a large heterogeneity (I^2^ = 51%). In the observational studies, the pooling of three studies [55,56,59] showed no significant effects improving quality of life ((mean = 5.595 95%, CI −21.347; 32.537) with important heterogeneity (I^2^ = 99%) (Figure 3). The leave-one-out analysis showed that Chikhaine et al.’s [55] was the study with the most influence in the reduction of effect (Figure 4).

When the subgroup analysis was performed (Figure 5), the RCT of Li et al. [50] showed a significant effect on improving physical component of the SF-12 after PR (Hedge´s g = 0.537 95% CI 0.159; 0.916, Z = 2.785, *p* = 0.005). In the observational studies, all the studies showed significant effects. Hayden et al. [56] found a reduction in EQ-5D-5L (mean = −2.42, 95% CI −2.94; −1.90), as did Chikhaine et al. [55], with a decrease in the SGRQ (mean = −14.90, 95% CI −21.86; −7.94). Additionally, Hayden et al. [56] also found increases in the EQ-5D-5L–VAS (mean = 18.04, 95% CI 15.28; 20.80), and Abodonya et al. [59] found increases in the EQ-5D-3L-VAS (mean 20.80, 95% CI 18.26; 23.34).

#### 3.4.4. Publication Bias of Included Studies

The Begg and Eggers tests are significant in the dyspnea and physical performance RCTs and, in the case of the Egger test, also significant in the physical performance of the observational studies, indicating the presence of a publication bias (Table 5).

**Figure 6:**
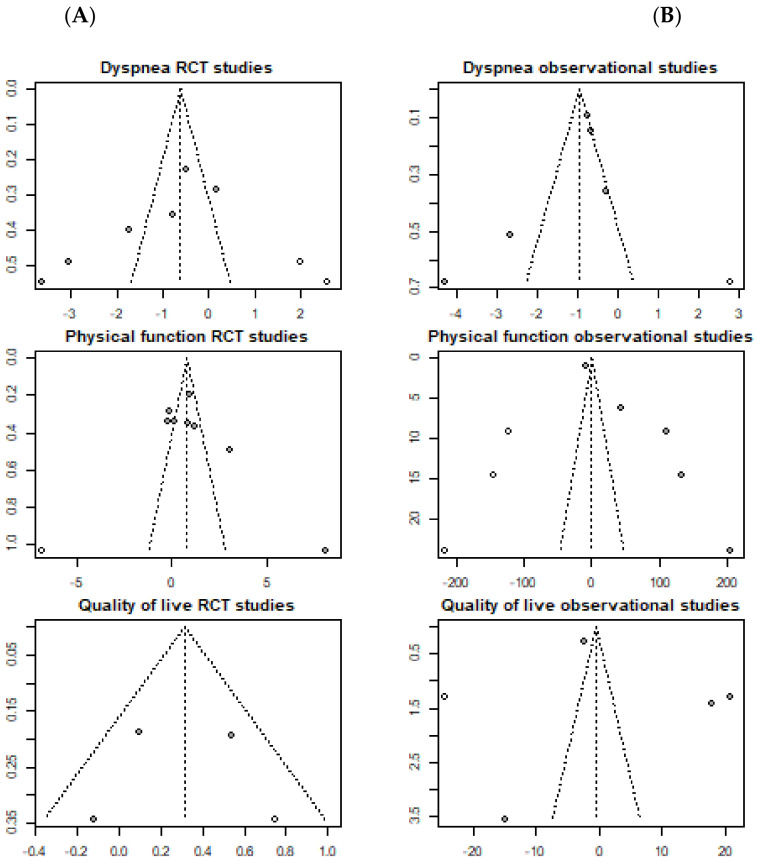
Publication bias of included studies on the effect of Pulmonary Rehabilitation in post-acute patients with COVID-19 related to physical function, dyspnea, and quality of life. (**A**) RCTs assessed; (**B**) Observational studies assessed.

The funnel plots show how most of the values are outside the significance bands, with an asymmetric distribution, which once again indicates the existence of a publication bias (Figure 6).

## 4. Discussion

The main objective of this systematic review and meta-analysis was to synthesize the evidence of the effectiveness of pulmonary rehabilitation in post-acute COVID-19 patients. Dyspnea, physical function, and quality of life were the main included outcomes, so they were our focus in the present review. All the included studies found improvements in dyspnea, physical function, and quality of life after pulmonary rehabilitation in patients with post-acute COVID-19. A very low quality of evidence and weak in favor GRADE suggests that pulmonary rehabilitation improves dyspnea, physical function, and quality of life in patients with post-acute COVID-19.

However, the meta-analysis revealed that, when all the studies were pooled, PR had no significant effect improving outcomes when compared to the control group and, in the observational studies, no significant effect was observed after PR in the patients with post-acute COVID-19, which is in contrast with other published meta-analyses [60,61] (Figure 3). Clearly, this data was biased due to the important heterogeneity across the studies. This heterogeneity was based on the measurement tools, as different studies used different scales. For example, when quality of life was pooled in the observational studies, the study of Chikhaine et al. [55] used the SGRQ to assess quality of life, and higher scores were related to a worse quality of life, whereas the study of Hayden et al. [56] used the EQ-5D-5L–VAS, in which the higher scores were related to a better quality of life, so pooling these data resulted in no significant effects after PR with a high heterogeneity.

To deal with this heterogeneity issue, an exploratory meta-analysis of the subgroup analyses was performed according to the different scales used to measure the main outcomes. When analyzing dyspnea, statistically significant differences were found in individual RCTs measured with the MD-12 and mMRC [49,50,54], with higher dyspnea levels in the control group after treatment, and in the observational studies, which measured dyspnea with the NRS, mMRC or DSI [56,59] (Figure 5). When physical function was analyzed by subgroup, significant effects were found in the observational studies, improving the 6MWD and 1-min STS after PR [55,56,57,58], while no significant effects were found in the treatment group compared to the control when evaluating the RCTs [49,50,51,53,54] in the subgroup analysis, also adding the TUG, SPPB and 30STS. Finally, the RCTs subgroup analysis of quality of life revealed significant improvements in the treatment group compared to the control in physical function when the SF-12 was used [50], and all the observational studies revealed significant differences after PR when assessed separately with the different measurement tools.

Although pooling resulted in all the data results being not significant due to the high heterogeneity, when analyzing them separately by subgroup, significant differences were found. Improvements in dyspnea, physical function, and quality of life were found after PR in patients with post-acute COVID-19. These results were in line with other previous studies, which conclude that PR helps in restoring lung function and improves physical function and quality of life [22,60,61,62].

Pulmonary rehabilitation has been demonstrated to be a safe and effective therapy in patients with post-acute COVID-19 and, due to the pandemic context, telerehabilitation programs have been postulated as available therapeutic tools. For example, Vieira et al. [28] found in their review (containing several of our included studies) that telerehabilitation improved variables, such as physical performance or dyspnea, in patients with COVID-19, although it included patients with post-acute and long COVID-19, and the quality of evidence was rated as low, highlighting that more research is needed to draw solid conclusions about the telerehabilitation efficacy.

Two years later, knowledge about the pandemic has improved considerably. Therefore, understanding the presence and origin of potential sequelae experienced by patients after COVID-19 should be an emerging priority for researchers and clinicians [6,18]. By addressing these sequelae, early exercise and rehabilitation protocols applied during the patient’s hospitalization and after discharge may help to improve musculoskeletal pain symptoms and prevent functional deterioration [17,18]. Physical activity through multicomponent programs generates an increase in function and decreases weakness in patients infected by COVID-19, preventing and reversing functional deterioration, among other comorbidities [17,18].

### 4.1. Future Directions

The qualitative analysis of the included studies shows that RP is effective, but it is necessary to homogenize the measurement instruments and intervention protocols.

It is recommended to develop randomized controlled clinical trials using similar interventions and outcomes so that a more complete and homogeneous meta-analysis can be developed.

### 4.2. Limitations

This review has some limitations. The main limitation of this meta-analysis lies in the heterogeneity of the included studies, which use tests with very different scales and, in some cases, with inverse scores, which makes data interpretation difficult. However, an exploratory meta-analysis was performed to evaluate, by subgroups, the efficacy of PR and positive results were found in favor of PR. Nonetheless, future studies should use the same scale to avoid this high heterogeneity. Second, we established linguistic filters, which is not recommended and may miss some articles in the process. Third, we focused on dyspnea, excluding other lung function parameters such as forced expiratory volume (FEV) or peak expiratory flow (PEF), which could have been interesting to include. Future reviews should include these parameters to assess the efficacy of PR in all lung function-related outcomes. Fourth, it should be noted that the experimental studies included had a significant bias in allocation concealment during the development of the entire intervention, with participant/therapist/assessor-blinding being the lowest-scoring item.

## 5. Conclusions

From a qualitative analysis point of view, PR is effective in improving physical function, reducing dyspnea, and improving quality of life in patients with post-acute COVID-19.

A very low quality of evidence and weak in favor GRADE suggests that pulmonary rehabilitation improves dyspnea, physical function, and quality of life in patients with post-acute COVID-19.

However, an exploratory meta-analysis was performed to evaluate, by subgroups, the efficacy of PR, and positive results were found in favor of PR.

## Figures and Tables

**Table 1:** Characteristics of the studies included in the systematic review.

Author (Year)	Study Design	Population	Sample Size	Intervention	Control Group	Outcomes	Results
González-Gerez et al. (2021) [49]	RCT	Adults mild-moderate acute COVID-19	N = 38	N = 19 (40.79 y/o, ±9.84; 47.4% F) Breathing exercises once a day, 7 days at home, telematic.	N = 19 (40.32 y/o, ±12.53; 42.1% F) Usual care	Physical function (6MWD/30STS) Dyspnea (MD12/BS)	Patients in intervention group improved dyspnea (MD12 and BS *p* < 0.001), 30STS (*p* = 0.001) and 6MWT (*p* = 0.006) after intervention compared to baseline. Patients in control group did not show statistically differences after intervention in any measured outcome compared to baseline. Between groups comparison, intervention group improved dyspnea (MD12 and BS *p* < 0.001), 30STS (*p* = 0.001) and 6MWT (*p* = 0.007) with differences compared to control.
Li et al. (2021) [50]	RCT	Adults with moderate dyspnea associated to COVID-19	N = 119 (55.46% F) Mean age 50.61 ± 10.98	N = 59 Breathing exercises Aerobic exercise Strength exercise 3–4 sessions per week, during 6 weeks	N = 60 Educational instructions at baseline	Physical function (6MWD) Dyspnea (mMRC) Quality of life (SF-12)	6MWD improved in both groups, but intervention group improved with statistically differences (*p* < 0.001). Quality of life improved in both groups, but intervention group improved with statistically differences in physical component (*p* = 0.004). Differences in mental component were not statistically significant (*p* = 0.116). Dyspnea improved in both groups, but intervention group improved with statistically differences (*p* = 0.001).
Rodríguez-Blanco et al. (2021) [51]	RCT	Adults mild-moderate acute COVID-19	N = 36	N = 18 (50% F) Age: 39.39 (±11.74) Resistance training once a day, for 7 days	N = 18 (55.5% F) Age: 41.33 (±12.13) Usual care	Physical function (6MWD/30STS) Dyspnea (MBS)	Patients in intervention group improved with statistically differences 6MWT (*p* = 0.016), 30STS (*p* = 0.011) and dyspnea (*p* < 0.001) compared to baseline, while control group improved 30STS (*p* = 0.026) and not 6MWT (*p* = 0.993) compared to baseline. Between groups comparison, intervention group improved 6MWT with statistically differences compared to usual care (*p* = 0.026) as well as 30STS (*p* = 0.001) and dyspnea (*p* < 0.001).
Bargahi et al. (2021) [52]	RCT	Adults with COVID-19 associated dyspnea and SpO_2_ < 94%.	N = 80 (38.75% F)	N = 40 Age: 57.1 (±18.7) Respiratory training 5 sets of 5 repetitions each day, for 3 days	N = 40 Age: 58 (±17.13) Usual care	Dyspnea (MBS)	Dyspnea improved after treatment in intervention group compared to control at rest (*p* = 0.007) and after walking 50 m (*p* = 0.017)
Fereydounnia et al. (2022) [53]	RCT	Adults with acute COVID-19 and oxygen therapy	N = 50 (42% F)	N = 25 Age: 49.44 (±14.78) Myofascial release therapy + Respiratory physical therapy, 3 times per week for 1 week	N = 25 Age: 45 (±12.75) Respiratory physical therapy, 3 times per week for 1 week	Dyspnea (MBS) Physical function (6MWD)	Statistically differences between groups were found in dyspnea perception (*p* < 0.01). 6MWD improved in control group but not in intervention.
Pehlivan et al. (2022) [54]	RCT	Adults with post-acute COVID-19	N = 34	N = 17 (18% F) Age: 50.76 (32–82) Education Aerobic Exercise Breathing Exercise Strength Exercise 3 days per week, for 6 weeks	N = 17 (35% F) Age: 43.24 (23–71) Exercises to be performed at home without supervision	Physical function (TUG/SPPB) Dyspnea (mMRC) Quality of life (SGRQ)	Both groups improved outcomes but only with differences intra-group in terms of dyspnea (*p* = 0.035), TUG (*p* = 0.005) and SGRQ (*p* = 0.002) in intervention group. No intra-group differences were found in control group. Between groups, only SGRQ improved with statistically differences in intervention group compared to control (*p* = 0.042).
Chikhaine et al. (2021) [55]	Observational	Adults with COVID-19 compared with Adults non-COVID-19 with respiratory failure	N = 42 (35.71% F)	N = 21 Age: 70.9 ± 10.6 Breathing Exercises Muscle strengthening Aerobic Exercise	N = 21 Age: 69.1 ± 9.4 Breathing Exercises Muscle strengthening Aerobic Exercise	Physical function (6MWD) Quality of life (SGRQ)	Pulmonary rehabilitation showed no differences in 6MWT improvements in COVID-19 patients compared to non-COVID-19 patients with respiratory failure (*p* < 0.001). Quality of life improved after treatment, but without statistically significant differences. However, both groups still showed impairment in respiratory function and physical performance at discharge.
Hayden et al. (2021) [56]	Observational	Adults post-acute to mild COVID-19	N = 108 (45.4% F) Age: 55.6 (±10.1)	Endurance training (3–5 sessions per week, 30–60 min) Breathing exercise (1 per week, 45 min) Physical Therapy Education (45 min) Psychosocial support Nutritional counseling Occupational therapy	No control	Dyspnea (NRS/mMRC) Physical function (6MWD) Quality of life (EQ-5D-5L)	Moderate to large pre-post changes for intensity in exertional dyspnea. 50% of patients improved with clinically differences (ES: 0.64 ± 0.23). 6MWD improved significantly with large effect size (ES: 1.36 ± 0.27). Quality of life improved significantly with high effect sizes (ES: 0.95 ± 0.26).
Martín et al. (2021) [57]	Observational	Adults with severe COVID-19	N = 48	N = 14 (21.4% F)Age: 60.8 (±10.4)Telerehabilitation program based on exercise, twice a week, for 6 weeks. 50 min per session.	N = 13 (53.8% F)Age: 61.9 (±10.7)Usual Care	Physical function (1min-STS)	At 3 months of follow-up, there were statistically differences favoring intervention group (*p* = 0.004) in terms of physical function improvements.
Büsching et al. (2021) [58]	Observational	Adults with pneumonia associated to COVID-19 compared to patients with other non-COVID-19 pneumonia	N = 102	N = 51 (25% F) Age: 65.8 (±11.7) Aerobic exercise Strength exercise Breathing exercise Relaxation techniques Psychological and nutritional counseling	N = 51 (55% F) Age: 69.8 (±9.6) Aerobic exercise Strength exercise Breathing exercise Relaxation techniques Psychological and nutritional counseling	Physical function (6MWD)	After intervention, both groups improved in 6MWD compared with baseline. Additionally, patients with pneumonia associated with COVID-19 improved more in 6MWD than patients with other causes pneumonia (*p* = 0.026).
Abodonya et al. (2021) [59]	Observational	Adults with post-acute COVID-19 compared to age-matched controls without COVID-19	N = 42	N = 21 (19% F) Age: 48.3 (±8.5) Breathing exercises Inspiratory muscle trainer, 2 sessions/day, 5 days/week, for 2 weeks.	N = 21 (23.8% F) Age: 47.8 (±9.2) Breathing exercises 2 times daily for 2 weeks	Dyspnea (DSI) Quality of life (EQ-5D-3L) Physical function (6MWD)	Intra-group analysis found statistically differences in intervention group in dyspnea (*p* = 0.039), quality of life (*p* < 0.001) and 6MWD (*p* < 0.001). However, there were improvements in control group, but without statistically differences. Between groups comparison found statistically differences favoring intervention group in dyspnea (*p* = 0.032), quality of life (*p* = 0.021) and 6MWD (*p* = 0.028) when compared to control.

Abbreviations: RCT (Randomized Controlled Trial); F (Female); 6MWD (6 Minute Walking Distance); 30STS (30 Seconds Sit To Stand test); MD12 (Multidimensional Dyspnea 12); mMRC (Modified Medical Research Council); SF-12 (Short Form 12); CI (Confidence Interval); MBS (Modified Borg Scale); TUG (Time Up and Go test); SPPB (Short Physical Performance Battery); SGRQ (Saint George Respiratory Questionnaire); BR (Borg Scale); NRS (Numeric Rating Scale); EQ-5D-5L (EuroQol-5D-5L); ES (Effect Size); 1 min-STS (One Minute Sit To Stand test); DSI (Dyspnea Severity Index).

**Table 2:** Risk of bias assessment of the RCTs using the Cochrane Risk of Bias Tool for assessing the risk of bias in randomized trials.

Author (Year)	Random Sequence Generation	Deviations from the Intended Interventions	Missing Outcome Data	Measurement of Outcomes	Selection of the Reported Results	Overall Risk of Bias
Gonzalez-Gerez et al. (2021) [49]	Low risk	Low risk	Low risk	Low risk	Low risk	Low risk
Li et al. (2021) [50]	Low risk	Low risk	Low risk	Low risk	Low risk	Low risk
Rodriguez-Blanco et al. (2021) [51]	Low risk	Low risk	Low risk	Low risk	Low risk	Low risk
Bargahi et al. (2021) [52]	Some concerns	Some concerns	Low risk	Low risk	Low risk	Some concerns
Fereydounnia et al. (202) [53]	Low risk	Some concerns	Some concerns	Low risk	Low risk	Some concerns
Pehvlian et al. (2022) [54]	Low risk	Some concerns	Some concerns	Some concerns	Low risk	Some concerns

**Table 3:** Newcastle–Ottawa Scale for assessing quality appraisal.

Study Name	Selection				Comparability	Exposure/Outcome			Total
	*1*	*2*	*3*	*4*	*1*	*1*	*2*	*3*	
Chikhaine et al., 2021 [55]	Y	Y	N	Y	Y N	N	Y	Y	6
Hayden et al., 2021 [56]	Y	Y	N	N	N N	N	Y	Y	4
Martín et al., 2021 [57]	Y	Y	N	N	Y N	Y	Y	Y	6
Büsching et al., 2021 [58]	Y	Y	N	Y	Y Y	Y	N	Y	7
Abodonya et al., 2021 [59]	Y	Y	Y	Y	Y Y	Y	N	Y	8

**Table 4:** Summary of findings for included studies using the GRADE quality of evidence assessment.

**Quality assessment of pulmonary rehabilitation improving dyspnea of post-acute COVID-19 patients**							
Number of studies (Subjects)	Risk of bias	Inconsistency	Indirectness	Imprecision	Publication Bias	Quality	Grade of recomendation
N = 7 (471)	Serious *	Serious ‡	Not serious	Serious ≠	Serious +	Very low quality	Weak in favor
**Quality assessment of pulmonary rehabilitation improving physical function of post-acute COVID-19 patients**							
Number of studies (Subjects)	Risk of bias	Inconsistency	Indirectness	Imprecision	Publication Bias	Quality	Grade of recommendation
N = 10 (598)	Serious *	Serious ‡	Not serious	Serious ≠	Serious +	Very low quality	Weak in favor
**Quality assessment of pulmonary rehabilitation improving quality of life of post-acute COVID-19 patients**							
Number of studies (Subjects)	Risk of bias	Inconsistency	Indirectness	Imprecision	Publication Bias	Quality	Grade of recommendation
N = 4 (303)	Serious *	Serious ‡	Not serious	Serious ≠	Serious +	Very Low quality	Weak in favor

* Most of studies with high risk of bias; ‡ Moderate to high heterogeneity between studies; ≠ Wide confidence intervals; + Presence of publication bias.

**Table 5:** Begg and Egger tests for publication bias.

Outcome and Study	Begg Test	Eggers Test
Dyspnea RCT	Kendall’s τ = −0.867, *p* = 0.017	t(4) = −5.193, *p* = 0.007
Physical function RCT	Kendall’s τ = 0.643, *p* = 0.031	t(6) = 5.976, *p* = 0.001
Quality of life RCT	Kendall’s τ = −0.333, *p* > 0.999	t(1) = −0.877, *p* = 0.542
Dyspnea OBS	Kendall’s τ = −0.4, *p* = 0.483	t(3) = −2.45, 0.092
Physical function OBS	Kendall’s τ = 0.4, *p* = 0.483	t(3) = 6.971, *p* = 0.006
Quality of life OBS	Kendall’s τ = 0, *p* > 0.999	t(2) = 0.824, *p* = 0.496

## Data Availability

The data presented in this study are available on request from the corresponding authors.
